# Comparison of the biocompatibility profiles of synthetic polysulfone and polyethersulfone dialysis membranes

**DOI:** 10.1093/ckj/sfag023

**Published:** 2026-01-30

**Authors:** Mohamed Belmouaz, Etienne Cogne, Florent Joly, Estelle Desport, Cecile Martin, David Lieurain, Fabien Duthe, Lisa Durocher, Antoine Thierry

**Affiliations:** Department of Nephrology, Poitiers University Hospital, Poitiers, France; Department of Nephrology, Poitiers University Hospital, Poitiers, France; Department of Nephrology, Poitiers University Hospital, Poitiers, France; Department of Nephrology, Poitiers University Hospital, Poitiers, France; Department of Nephrology, Poitiers University Hospital, Poitiers, France; Department of Nephrology, Poitiers University Hospital, Poitiers, France; Department of Nephrology, Poitiers University Hospital, Poitiers, France; Department of Biostatistics, Centre d’Investigation Clinique CIC 1402, Poitiers University Hospital, Poitiers, France; Department of Nephrology, Poitiers University Hospital, Poitiers, France

To the Editor,

Chronic inflammation and oxidative stress are major contributors to morbidity and mortality in haemodialysis (HD) patients [1]. The HD procedure itself may also promote these adverse effects [2]. The dialysis membrane represents the main site of blood exposure to non-biological material, the physicochemical characteristics of which may trigger various biological reactions and determine biocompatibility. Over the past decades, leucocyte, platelet, coagulation system and complement system activation have been described as reliable markers of biocompatibility [3, 4]. Moreover, repeated intradialytic contact of blood with artificial materials may result in continuous leucocyte and complement system activation, leading to the release of pro-inflammatory cytokines, oxidative stress markers, pro-inflammatory anaphylatoxins (C3a, C5a) and the membrane attack complex sC5b-9. These immune disturbances may contribute to long-term cardiovascular disease and mortality in HD patients [2].

Advances in membrane biocompatibility from cellulose-based (cuprophane) to synthetic membranes have substantially improved biocompatibility [2]. Synthetic high-flux (HF) membranes manufactured from sulfonyl group–containing polymers, such as polysulfone (PS) and polyethersulfone (PES), are now widely used in both HD and haemodiafiltration modes. However, given the wide range of commercially available PS and PES membranes, which differ in their manufacturing process (e.g. fibre geometry, surface charge, content of hydrophilic polyvinylpyrrolidone and sterilization methods), their biocompatibility equivalences remain uncertain.

We recently conducted a randomized crossover study [5] including 36 patients assigned to receive either medium-cut-off HD or super HF vitamin E–coated HD. Prior to randomization, all patients were treated with HF-HD for 3 months. Among the 36 patients, 14 were treated with the polynephron PES Elisio 21H dialyser (Nipro Europe, Zaventem, Belgium), 10 with the PS TS-2.1 SL (Meditor France, Toray Industries, Tokyo, Japan) and 12 with the PES V20-HF (Theradial France, Vital Healthcare, Kapar, Malaysia).

At baseline, serum levels of human interleukin 6 (IL-6), tumour necrosis factor-alpha (TNF-α), soluble TNF-α receptor 1 (sTNFR1), malondialdehyde (MDA), superoxide dismutase (SOD) activity, human oxidized low-density lipoprotein (ox-LDL), as well as leucocyte, polymorphonuclear neutrophil, monocyte and platelet counts were measured pre-, 15 min after initiation and post-dialysis.

In a secondary analysis, serum levels of C3a, sC5b-9 and C5a were measured at the same time points using an enzyme-linked immunosorbent assay kit (Novus Biologicals and R&D Systems Europe, Bio-Techne, Abingdon, UK).

**Table 1:** Comparison of the variation in biocompatibility parameters between three HF synthetic membranes.

Characteristics	PES (ELISIO 21H) membrane (*n* = 14)	PS (TSL 2.1-SL) membrane (*n* = 10)	PES (V20-HF) membrane (*n* = 12)	*P*-value
IL-6				
Pre-dialysis (pg/ml)	0.6 (0.1–1.5)	0.8 (0.3–2.2)	0.9 (0.0–2.8)	NP
After 15 min (pg/ml)	0.7 (0.1–1.1)	0.9 (0.5–2.1)	0.8 (0.4–3.7)	NP
Post-dialysis (pg/ml)	0.6 (0.1–1.5)	0.8 (0.3–2.2)	0.9 (0.0–2.8)	NP
RV (%) after 15 min	−0.1 (−15.0–24.9)	5.5 (−6.3–66.1)	11.0 (−4.2–50.7)	.590
RV (%) after 240 min	−11.1 (−47.0–462.4)	3.8 (−8.6–38.2)	18.1 (−12.2–81.8)	.932
TNF-α				
Pre-dialysis (pg/ml)	0.1 (0.1–0.1)	0.4 (0.1–3.4)	0.1 (0.1–1.5)	NP
After 15 min (pg/ml)	0.1 (0.1–2.3)	3.0 (1.1–6.7)	1.6 (0.1–21.0)	NP
Post-dialysis (pg/ml)	0.1 (0.1–0.1)	0.4 (0.1–3.4)	0.1 (0.1–1.5)	NP
RV (%) after 15 min	0.0 (0.0–0.0)	129.3 (−5.7–1605.5)	12.8 (0.0–2405.9)	.166
RV (%) after 240 min	−1.5 (−2.1 to −1.0)	−4.3 (−94.5 to −1.2)	−1.4 (−7.3 to −1.0)	.265
sTNFR1				
Pre-dialysis (ng/ml)	13.7 (10.0–15.4)	12.9 (10.5–18.0)	15.3 (12.9–17.4)	NP
After 15 min (ng/ml)	14.7 (11.3–16.3)	14.2 (10.8–18.8)	15.6 (14.4–18.4)	NP
Post-dialysis (ng/ml)	13.7 (10.0–15.4)	12.9 (10.5–18.0)	15.3 (12.9–17.4)	NP
RV (%) after 15 min	6.7 (4.3–12.7)	3.9 (2.3–6.8)	6.5 (3.9–12.6)	.548
RV (%) after 240 min	6.5 (−1.3–10.5)	3.4 (−7.2–11.0)	−9.0 (−10.9–2.0)	.063
ox-LDL				
Pre-dialysis (U/l)	45.4 (41.2–59.7)	46.3 (41.6–68.3)	59.8 (47.4–73.0)	NP
After 15 min (U/l)	48.9 (36.5–58.4)	47.4 (36.2–64.9)	58.9 (45.2–73.3)	NP
Post-dialysis (U/l)	45.4 (41.2–59.7)	46.3 (41.6–68.3)	59.8 (47.4–73.0)	NP
RV (%) after 15 min	−5.1 (−19.2–3.9)	−2.0 (−14.6–3.6)	−3.5 (−16.0–3.2)	.921
RV (%) after 240 min	12.4 (2.0–17.4)	12.4 (−0.2–27.3)	9.0 (−4.9–18.9)	.830
SOD activity				
Pre-dialysis (U/ml)	4.0 (3.6–8.2)	4.0 (3.2–5.1)	3.9 (3.2–5.3)	NP
After 15 min (U/ml)	4.3 (3.4–5.8)	4.2 (3.1–5.0)	4.6 (2.8–5.0)	NP
Post-dialysis (U/ml)	3.3 (2.6–5.0)	3.4 (2.8–4.1)	3.0 (2.4–4.1)	NP
RV (%) after 15 min	0.0 (−26.9–13.1)	−4.5 (−16.8–20.7)	−3.6 (−24.3–35.3)	.981
RV (%) after 240 min	−24.3 (−35.5 to −15.2)	−19.2 (−39.3 to 6.4)	−23.3 (−46.1 to −5.7)	.815
MDA				
Pre-dialysis (ng/ml)	305.2 (190.7–1277.4)	250.3 (137.1–416.7)	264.3 (213.8–359.6)	NP
After 15 min (ng/ml)	265.7 (159.1–1349.1)	359.2 (204.3–671.5)	323.2 (191.3–398.4)	NP
Post-dialysis (ng/ml)	305.2 (190.7–1277.4)	250.3 (137.1–416.7)	264.3 (213.8–359.6)	NP
RV (%) after 15 min	5.6 (−19.6–44.0)	21.6 (−11.5–71.1)	3.9 (−27.9–35.6)	.330
RV (%) after 240 min	0.3 (−33.9–37.5)	12.9 (−34.3–67.3)	−11.9 (−36.3–12.8)	.407
Leucocyte				
Pre-dialysis (10^9^ cells/l)	5.9 (5.3–7.2)	6.1 (5.3–6.5)	4.9 (4.3–5.9)	NP
After 15 min (10^9^ cells/l)	6.0 (5.0–6.2)	5.8 (4.6–6.0)	4.8 (4.2–5.6)	NP
Post-dialysis (10^9^ cells/l)	5.9 (5.3–7.2)	6.1 (5.3–6.5)	4.9 (4.3–5.9)	NP
RV (%) after 15 min	−8.1 (−15.6–0.0)	−10.3 (−14.8 to −3.1)	−5.2 (−9.3–0.4)	0.602
RV (%) after 240 min	−3.2 (−13.5–13.1)	−11.1 (−14.8 to −5.7)	−3.7 (−11.1–15.7)	0.319
Polymorphonuclear neutrophil				
Pre-dialysis (10^9^ cells/l)	4.3 (3.1–5.0)	3.5 (2.7–4.3)	3.1 (2.6–3.5)	NP
After 15 min (10^9^ cells/l)	4.0 (3.1–5.3)	3.1 (2.7–4.0)	3.0 (2.5–3.6)	NP
Post-dialysis (10^9^ cells/l)	4.3 (3.1–5.0)	3.5 (2.7–4.3)	3.1 (2.6–3.5)	NP
RV (%) after 15 min	−6.4 (−12.8 to −0.5)	−8.8 (−12.1 to −1.2)	−1.2 (−5.4–4.1)	.170
RV (%) after 240 min	−2.8 (−14.5–12.2)	−14.3 (−16.9 to −8.1)	3.3 (−10.3–21.1)	.103
Monocyte				
Pre-dialysis (10^9^ cells/l)	0.5 (0.5–0.7)	0.6 (0.4–0.8)	0.6 (0.5–0.7)	NP
After 15 min (10^9^ cells/l)	0.4 (0.3–0.6)	0.4 (0.3–0.6)	0.4 (0.4–0.6)	NP
Post-dialysis (10^9^ cells/l)	0.5 (0.5–0.7)	0.6 (0.4–0.8)	0.6 (0.5–0.7)	NP
RV (%) after 15 min	−23.7 (−38.0 to −10.4)	−23.5 (−28.9 to −17.3)	−19.6 (−25.4 to −10.6)	.734
RV (%) after 240 min	−1.8 (−14.4–23.4)	−9.8 (−17.9 to −2.3)	−12.5 (−25.5 to −6.5)	.549
Platelet				
Pre-dialysis (10^9^ cells/l)	193.5 (163.0–225.0)	185.0 (122.0–191.0)	170.0 (118.0–236.0)	NP
After 15 min (10^9^ cells/l)	190.0 (172.0–234.0)	175.0 (133.0–187.0)	161.5 (119.5–203.5)	NP
Post-dialysis (10^9^ cells/l)	193.5 (163.0–225.0)	185.0 (122.0–191.0)	170.0 (118.0–236.0)	NP
RV (%) after 15 min	−2.6 (−6.2–2.4)	−2.7 (−8.9 to −0.8)	−4.7 (−12.9–1.9)	.620
RV (%) after 240 min	1.4 (−3.6–7.8)	1.3 (−0.5–5.7)	−2.0 (−10.2–16.2)	.795
C3a				
Pre-dialysis (ng/ml)	30.7 (26.7–43.6)	34.5 (30.2–53.7)	39.5 (28.4–73.5)	NP
After 15 min (ng/ml)	32.7 (20.5–37.6)	29.0 (21.8–39.4)	36.1 (11.1–51.2)	NP
Post-dialysis (ng/ml)	30.7 (26.7–43.6)	34.5 (30.2–53.7)	39.5 (28.4–73.5)	NP
RV (%) after 15 min	−12.9 (−16.9–12.4)	−20.1 (−26.6–0.0)	−43.4 (−57.8 to −4.4)	.075
RV (%) after 240 min	6.0 (−8.2–16.5)	−5.6 (−15.3–7.7)	−10.9 (−28.4–9.4)	.326
C5a				
Pre-dialysis (ng/ml)	73.1 (65.3–111.3)	97.3 (85.7–112.3)	86.2 (66.6–111.0)	NP
After 15 min (ng/ml)	59.0 (53.5–84.3)	75.2 (63.9–80.0)	65.9 (52.5–85.9)	NP
Post-dialysis (ng/ml)	73.1 (65.3–111.3)	97.3 (85.7–112.3)	86.2 (66.6–111.0)	NP
RV (%) after 15 min	−17.7 (−29.9 to −13.4)	−30.2 (−41.8 to −8.4)	−20.7 (−37.5 to −4.3)	.913
RV (%) after 240 min	3.0 (−6.2–11.4)	−11.4 (−23.2–10.2)	−1.7 (−13.6–11.3)	.312
sC5b-9				
Pre-dialysis (µg/ml)	15.2 (11.6–19.4)	17.3 (15.4–23.2)	19.6 (5.5–23.2)	NP
After 15 min (µg/ml)	10.5 (5.6–18.0)	11.3 (8.5–13.9)	10.1 (4.0–18.9)	NP
Post-dialysis (µg/ml)	15.2 (11.6–19.4)	17.3 (15.4–23.2)	19.6 (5.5–23.2)	NP
RV (%) after 15 min	−4.9 (−52.0–21.8)	−32.3 (−52.7–40.2)	−24.8 (−42.7–0.0)	.809
RV (%) after 240 min	22.7 (−15.3–66.1)	−5.9 (−25.7–9.9)	8.2 (−15.8–51.7)	.276

NP: not performed.

Data are expressed as median (IQR).

Patients’ characteristics, dialysis parameters and membranes characteristics are reported in the supplementary material. Sampling procedures and calculation methods have been previously described [5]. Quantitative variables are presented as median and interquartile range (IQR) and were compared using the Kruskal–Wallis test.

The three synthetic membranes induced similar relative variation (RV) 15 min after dialysis initiation and post-dialysis of IL-6, TNF-α, sTNFR1, MDA, SOD, ox-LDL, C3a, C5a, sC5b-9 and leucocyte and platelet counts (Table 1).

The evaluation of dialysis membrane biocompatibility remains challenging because of the complexity and interaction of multiple factors, including dialysis procedures, patient comorbidities and underlying pathological conditions. Furthermore, the most appropriate methods for assessing membrane biocompatibility remain to be defined in a clinical setting. Although previous studies have reported biocompatibility differences among commercially available PS and PES [2, 6], our findings indicate comparable effects on the fluctuation of leucocyte, platelet, complement anaphylatoxins, inflammatory and oxidative stress parameters among the three synthetic membranes evaluated.

Several limitations of this study should be acknowledged, including lack of randomisation, absence of an internal control, small sample size, methodological constraints, limited scope of biocompatibility markers and patient-related clinical factors. Nevertheless, the three synthetic membranes, which have a nearly similar gamma-ray sterilisation method, appear to have comparable variations in biocompatibility parameters.

In conclusion, the present study suggests that the evaluated biocompatibility parameters are comparable among the three synthetic membranes. From a clinical perspective, these membranes appear to be equivalent with regard to the biocompatibility markers assessed.

## Supplementary Material

sfag023_Supplemental_File
