# Supplementary material for: Comparison of the biocompatibility profiles of synthetic polysulfone and polyethersulfone dialysis membranes
Source: Clin Kidney J. 2026 Jan 30;19(3):sfag023. doi: 10.1093/ckj/sfag023 (PMC13006871; doi:10.1093/ckj/sfag023)
Supplement: sfag023_Supplemental_File [file sfag023_supplemental_file.docx]

**Supplementary material**

**Supplementary material table S1. Characteristics of high-flux dialysis membranes**

| **Dialyzer**  **Number of patients (n=36)** | **Membrane polymer** | **Membrane type** | **Fiber inner diameter (μm)** | **Membrane area (m²)** | **Membrane wall thickness (μm)** | **UFC (ml/h/mmHg)** | **Beta2-m SC** | **Albumin SC** | **Sterilization** |
| --- | --- | --- | --- | --- | --- | --- | --- | --- | --- |
| **TS-2.1 SL™**  **n=10 (28%)** | **PS** | **High-flux** | **200** | **2.1** | **40** | **52** | **0.8** | **0.003** | **Gamma-Ray** |
| **V-20 HF™**  **n=12 (33%)** | **PES** | **High-flux** | **200** | **2** | **40** | **66** | **≥ 0.7** | **≤ 0.001** | **Gamma-Ray** |
| **Elisio**  **21H™**  **n=14 (39%)** | **Polynephron /PES** | **High-flux** | **200** | **2.1** | **40** | **82** | **0.8** | **0.002** | **Dry Gamma** |

Abbreviations: PS : polysulfone, PES: polyethersulfone, UFC: ultrafiltration coefficient, Beta2-m: beta2microglobulin, SC: Sieving coefficient

**Supplementary material Table S2 – Characteristics of the study population**

|  | **All  (n=36)** | **PES (ELISIO 21H) membrane (n=14)** | **PS (TSL 2.1-SL) membrane (n=10)** | **PES (V20-HF) membrane (n=12)** | ***P* value** |
| --- | --- | --- | --- | --- | --- |
| Age (years) | 73.5 (64.0-79.0) | 74.5 (66.0-78.0) | 74.5 (63.0-80.0) | 67.5 (62.5-79.5) | 0.750 |
| Gender (female/male), n (%) | 17 (47%) / 19 (53%) | 9 (64%) / 5 (36%) | 1 (10%) / 9 (90%) | 7 (58%) / 5 (42%) | 0.019 |
| Body height (cm) | 164.5 (159.5-172.5) | 163.0 (159.0-171.0) | 171.5 (160.0-175.0) | 162.5 (159.0-168.5) | 0.267 |
| Body weight (kg) | 75.3 (56.2-86.0) | 75.3 (60.0-86.1) | 72.2 (63.5-85.5) | 75.2 (51.5-90.0) | 0.836 |
| BMI (kg/m²) | 27.2 (20.9-30.0) | 28.1 (24.0-30.6) | 26.8 (20.7-28.6) | 27.6 (20.3-31.8) | 0.445 |
| Residual urine output, n (%) |  |  |  |  | 0.481 |
| ≤ 200 ml/day | 25 (69%) | 8 (57%) | 8 (80%) | 9 (75%) |  |
| ≥ 300 and < 500 ml/day | 11 (31%) | 6 (43%) | 2 (20%) | 3 (25%) |  |
| CKD cause, n (%) |  |  |  |  | 0.039 |
| Diabetic nephropathy | 3 (8%) | 1 (7%) | 0 (0%) | 2 (17%) |  |
| Glomerulopathy | 5 (14%) | 0 (0%) | 2 (20%) | 3 (25%) |  |
| Interstitial nephritis | 3 (8%) | 0 (0%) | 2 (20%) | 1 (8%) |  |
| Nephrosclerosis | 9 (25%) | 3 (21%) | 2 (20%) | 4 (33%) |  |
| Polycystic kidney disease | 5 (14%) | 2 (14%) | 1 (10%) | 2 (17%) |  |
| Unknown | 11 (31%) | 8 (57%) | 3 (30%) | 0 (0%) |  |
| Comorbidities, n (%) |  |  |  |  |  |
| Diabetes | 12 (33%) | 6 (43%) | 2 (20%) | 4 (33%) | 0.551 |
| Hypertension | 31 (86%) | 11 (79%) | 8 (80%) | 12 (100%) | 0.232 |
| Dyslipidemia | 18 (50%) | 7 (50%) | 5 (50%) | 6 (50%) | 1.000 |
| Heart disease | 4 (11%) | 0 (0%) | 4 (40%) | 0 (0%) | 0.004 |
| Cerebrovascular disease | 4 (11%) | 1 (7%) | 0 (0%) | 3 (25%) | 0.222 |
| Peripheral vascular disease | 8 (22%) | 4 (29%) | 2 (20%) | 2 (17%) | 0.880 |
| Vascular access, n (%) |  |  |  |  | 0.099 |
| Native arteriovenous fistula | 22 (61%) | 9 (75%) | 8 (80%) | 5 (42%) |  |
| Permanent tunneled catheter | 14 (39%) | 5 (35%) | 2 (20%) | 7 (58%) |  |

**Supplementary material Table S3 – Comparison of dialysis parameters**

|  | **PES (ELISIO 21H) membrane (n=14)** | **PS (TSL 2.1-SL) membrane (n=10)** | **PES (V20-HF) membrane (n=12)** | ***P* value** |
| --- | --- | --- | --- | --- |
| Blood flow rate (mL/min) | 300.0 (297.5-300.0) | 325.0 (300.0-338.0) | 300.0 (300.0-330.0) | 0.257 |
| Dialysate flow rate (mL/min) | 500 | 500 | 500 | NS |
| Blood volume processed (L) | 70.4 (68.4-72.5) | 77.4 (73.5-80.2) | 71.8 (68.5-72.1) | 0.142 |
| Ultrafiltration (mL) | 2370.0 (1700.0-2580.0) | 2025.0 (1700.0-2800.0) | 2130.0 (1600.0-2350.0) | 0.706 |
| Kt/V monitor | 1.4 (1.2-1.5) | 1.4 (1.2-1.6) | 1.5 (1.1-1.8) | 0.889 |

NS : not significant

Statistical analysis

Quantitative variables were presented as median and interquartile range and compared using a Kruskal-Wallis test. Qualitative variables were presented as numbers and percentages, then compared using a Chi-squared test or a Fisher exact test in case of expected value less than 5.
